# Dietary curcumin enhances insulin clearance in diet-induced obese mice via regulation of hepatic PI3K-AKT axis and IDE, and preservation of islet integrity

**DOI:** 10.1186/s12986-019-0377-0

**Published:** 2019-07-25

**Authors:** Yoo Kim, Michael Rouse, Isabel González-Mariscal, Josephine M. Egan, Jennifer F. O’Connell

**Affiliations:** Laboratory of Clinical Investigation, National Institute on Aging, National Institutes of Health, 251 Bayview Blvd, Suite 100, Baltimore, MD 21224 USA

**Keywords:** Curcumin, Insulin sensitivity, Phosphoinositide 3-kinase (PI3K), Insulin degrading enzyme (IDE), Hepatic insulin signaling pathway, Islet integrity

## Abstract

**Background:**

Although type 2 diabetes mellitus (T2DM) is primarily characterized by sustained high levels of circulating glucose, other factors, such as obesity, chronic inflammation, fatty liver, and islet dysfunction significantly contribute to the development of this disease. To date, curcumin (CUR), a natural polyphenol and primary component of turmeric, shows putative therapeutic properties such as reducing the incidence of obesity-related diseases in mice. However, the mechanism by which CUR regulates insulin levels remains unclear.

**Methods:**

This study investigates how dietary CUR improves insulin clearance and maintains a proper range of circulating insulin level in the diet-induced obesity (DIO) mouse model. Male C57BL/6 J mice were fed a control, a high fat/high sugar (HFS) or a HFS diet containing 0.4% (w/w) curcumin (HFS + CUR) (*N* = 16 per group) for 16 weeks.

**Results:**

Mice given HFS + CUR had reduced body weight and fat accumulation in the liver and had lower blood insulin levels under fasting conditions compared to mice on HFS alone, resulting from significantly improved insulin clearance via upregulation of hepatic insulin-degrading enzyme (IDE). We also observed restoration of phosphoinositide 3-kinase (PI3K), especially class Ia catalytic subunits, p110α and p110β, and class Ib regulatory subunit, p101, and phosphorylated protein kinase B (AKT) expression levels in liver on HFS + CUR diet. Additionally, HFS + CUR fed mice had significantly smaller islets of Langerhans and increased glucagon contents compared to HFS fed mice, indicating less secretion of insulin in pancreas. The expression of thioredoxin interacting protein (TXNIP), a pro-oxidant and pro-apoptotic protein, was significantly elevated in mouse and human islets cultured under HFS mimicking conditions, which was mitigated by CUR treatment.

**Conclusions:**

CUR supplementation in obese subjects may alleviate the burden imposed by HFS diets. Our data indicate administration of dietary CUR reinstates PI3K, AKT and IDE levels in obese mice. Additionally, CUR treatment preserves islet integrity by downregulation of TXNIP transcription levels. Therefore, dietary CUR may have the potential to serve as a novel therapeutic agent to address the underlying links of obesity and T2DM.

## Background

In populations where “Westernized” diets high in fat and sugar are prominent, one of the primary health problems that arises is obesity. Dyslipidemia, insulin resistance, and diabetes are just a few metabolic diseases that are commonly associated with the development of obesity [1]. In Type 2 diabetes mellitus (T2DM), pancreatic insulin secretion and insulin sensitivity become impaired, compounded by excessive gluconeogenesis, which leads to chronic dysglycemia [2]. Furthermore, in both obesity and T2DM, the levels of plasma free fatty acids (FFA) are elevated, which is conducive to insulin resistance and impaired insulin secretion [3]. Consequently, there remains a pressing need to identify novel therapeutic targets and develop effective treatments for T2DM by controlling circulating insulin levels.

In attempts to uncover better treatment options for T2DM, we have been investigating the therapeutic potential of nutraceuticals. One such compound is Curcumin (CUR), the most active component of the spice turmeric. Not only is CUR considered to be relatively inexpensive and safe, but previous studies found that CUR administration promotes weight loss, thus reducing the incidence of obesity-related diseases. We discovered that in vitro, CUR directly inhibits phosphodiesterase activity, thus enhancing insulin secretion and cAMP production in mouse pancreatic β-cells and primary human islets [4]. Additionally, some beneficial effects, such as reduced glycemia and hyperlipidemia, have been described in CUR fed diet-induced or genetically modified diabetic animal models [5]. Recently, several studies have revealed a putative molecular mechanism of CUR associated with improved insulin resistance. For example, CUR activates insulin receptor (IR), insulin receptor substrate 1 (IRS1) through tyrosine-phosphorylation, and downstream markers like protein kinase B (AKT) in the insulin signaling pathway [6–8]. Also, CUR regulates the sterol regulatory element-binding proteins (SREBPs) pathway or inhibits inflammation and oxidative stress via nuclear factor kappa B (NF-κB) and c-Jun NH2 terminal kinase (JNK), resulting in improvement of insulin sensitivity [9–11]. However, in terms of regulation of circulating insulin levels, the underlying mechanism and the relationship between insulin sensitivity and islet integrity by CUR treatment are still unclear.

We hypothesized that when given in conjunction with a HFS diet (diet-induced obesity (DIO) mouse model), CUR may prevent pancreatic islet changes and promote insulin signaling, ultimately regulating circulating insulin levels. Our results showed that CUR has pleiotropic effects in improving insulin clearance in liver, mediating the hepatic insulin pathway signaling, and reducing pancreatic oxidative stress in mice administered a HFS diet.

## Methods

### Animals and diets

Male C57BL/6 J mice (6 weeks) were purchased from The Jackson Laboratory (Bar Harbor, ME). All animals were housed at the National Institutes on Aging (NIA) which is fully accredited by the American Association for Accreditation of Laboratory Animal Care, and all procedures were approved by the Animal Care and Use Committee of the NIA Intramural Program. Animals were acclimated to the facility for 1 week before baseline assessments were performed. During this time, all mice were maintained on standard NIH chow (Teklad Global Rodent Diet, Envigo, Indianapolis, IN). After baseline assessment, they were randomized into one of three groups: a normal chow diet (Control; *n* = 16), a high fat/high sugar diet (HFS; *n* = 16) or a HFS diet containing 0.4% (w/w) of curcumin (HFS + CUR; *n* = 16) for 16 weeks. The dose of CUR in this study was determined based on the previous clinical studies [12]. This amount of CUR is equivalent to 2 g/day for a 60 kg adult calculated with equivalent surface area dosage conversion method [13]. The composition of the diets is shown in Table 1 and all diets were specially formulated by Dyets Inc. (Bethlehem, PA). Curcumin for diets was purchased from Sigma-Aldrich (St. Louis, MO). Mice were allowed ad libitum access to food and water throughout the study. The average body weight and food and water consumption were calculated weekly for 16 weeks.

**Table 1:** Mouse Diet Composition (grams/kg)

Ingredient	Control^a^	HFS^a^	HFS + CUR^a^
High Nitogen Casein	200	200	200
L-Cystine	3	3	3
Sucrose	90	90	90
Cornstarch	398.562	100.46	100.46
Dyetrose	132	160	160
Dextrose	0	160	156.1
Soybean oil	70	70	70
*tert*-Butylhydroquinone	0.014	0.014	0.014
Whole Butter (18% Water)	0	150	150
Ethoxyquin	0.024	0.024	0.024
Lard	0	20	20
Cellulose	50	50	50
Mineral mix #210025	35	35	35
Vitamin mix #310025	10	10	10
Supplement #410750	10	10	10
Choline Chloride	1.4	1.4	1.4
Red-Dye (roughly 31 g of water will evaporate)	0	0.1	0
Curcumin (roughly 31 g of water will evaporate)	0	0	4
Total	1,000	1,000	1,000

^a^*Control* Control diet, *HFS* High fat high sugar diet, *CUR* Curcumin

### Glucose metabolism experiments

Mice were fasted overnight and given free access to water. Blood samples were collected from tail veins at baseline and every 4 weeks until the end of the study for glucose and insulin measurements. For intraperitoneal glucose tolerance tests (IPGTT), glucose levels in blood were monitored and blood samples were collected from overnight fasted mice at 0, 15, 30, 60, and 90 min time points following administration of 1 g glucose/kg body weight at week 16. For insulin tolerance tests (ITT), blood glucose levels were measured from tail veins of 4 h fasted mice at 0, 15, 30, 60, and 90 min time points following intraperitoneal administration of recombinant human insulin from Novo Nordisk (1 U insulin/kg body weight). Blood glucose was measured using a hand-held glucometer (AccuChek, Roche Diabetes Care Inc., Indianapolis, IN). Blood plasma was used to measure insulin levels using an Ultra-Sensitive Mouse Insulin ELISA kit (Crystal Chemical Inc., Downers Grove, IL). The homeostasis model for insulin resistance (HOMA-IR) was calculated as fasting blood glucose (mg/dL) × fasting plasma insulin (μU/mL) / 405 [14].

### Western blot analysis

Mouse liver tissue samples were homogenized in tissue lysis buffer (25 mM Tris (pH 7.4), 2 mM Na_3_VO_4_, 10 mM NaF, 10 mM Na_4_P_2_O_7_, 1 mM EGTA, 1 mM EDTA, and 1% NP-40) containing phosphatase and protease inhibitor cocktails in the OMNI BeadRuptor 24 (Omni-Inc, Kennesaw, GA). Protein was quantified using a BCA Assay (ThermoFisher Scientific, Rockford, IL) and then protein loading samples were resolved in SDS-PAGE under reducing conditions and transferred to polyvinylidene fluoride (PVDF) membrane. Membranes were blocked in blocking reagent (LI-COR, Lincoln, NE) assay system for 1 h at room temperature and incubated with primary antibodies overnight at 4 °C as follows: IDE (1:1000) from ThermoFisher Scientific (Waltham, MA), GAPDH (1:3000), AKT (1:1000), p-AKT^Ser473^ (1:1000), β-tubulin (1:3000), PP2A B subunit (1:1000), p85 (1:1000), p110α (1:1000), p110β (1:1000), p110γ (1:1000), p110δ (1:1000), p110 class III (1:1000) and p101 (1:1000) from Cell Signaling Technology (Danvers, MA), and PHLPP1 (1:1000) and Gβ (1:1000) from EMD Millipore (Billerica, MA) and PP2A A subunit (1:1000) from BD Biosciences and p-PP2A A/B subunit (1:1000) from Santa Cruz Biotechnology (Dallas, TX). Membranes were washed with TBS-T and the appropriate secondary antibody (1:20000, ThermoScientific, Rockford, IL) was added in blocking reagent for 1 h at room temperature. Membranes were washed three times with TBS-T and developed using a chemiluminescence assay system. Western blot images were scanned, saved as Tiff files, inverted, and integrated density was analyzed using ImageJ software. Phosphorylated protein levels were normalized to the respective total protein levels.

### Oil red O staining

At the end of the study, livers from Control, HFS, and HFS + CUR mice were fixed in 4% paraformaldehyde overnight, cryoprotected in 30% sucrose, embedded in O.C.T. compound, frozen, and then sectioned on a cryostat (8 μm). Tissue sections were stained with freshly prepared Oil Red O (Sigma-Aldrich) for 5 min at room temperature and then washed with dH_2_O for 20 min. Slides were mounted with aqueous mounting medium and imaged at 20X on an Olympus IX51 inverted microscope (Waltham, MA). Quantitative analysis was based on at least 140 images per group (*n* = 8 per group) using ImageJ software after converting the images to 8-bit grayscale images.

### Immunofluorescence staining

At the end of the study, pancreata from control, HFS, and HFS + CUR fed mice were fixed in 4% paraformaldehyde overnight, embedded in paraffin blocks, and sectioned (5 μm). After deparaffinization, tissue sections were treated with antigen unmasking solution (Vector Laboratories, Burlingame, CA), washed, permeabilized, blocked, and primary antibodies were added overnight at 4 °C as follows: insulin (1:100; Dako, Carpinteria, CA); glucagon (1:500; Sigma-Aldrich, Saint Louis, MO). After washing, sections were incubated with fluorescently labeled secondary antibodies (1:1000; Alexa Fluor 488 or Alexa Fluor 647, ThermoFisher Scientific) and DAPI (1:5000; ThermoFisher Scientific) for nuclear staining. Images were obtained on a Zeiss LSM-710 confocal microscope (Jena, Germany) the 40X oil objective. Islet size was determined using Fiji-Image J software (NIH). Quantitative analysis was based on at least 150 images per group (*n* = 8 per group).

### DAB staining

Paraffin-embedded pancreata were prepared as described above. After treatment with antigen unmasking solution, basal peroxidase activity was blocked with 3% H_2_O_2_. Tissue sections were then washed with PBS, permeabilized with Triton X-100, blocked with 5% BSA, and primary TXNIP antibody (1:200, Abcam, Cambridge, MA) was added overnight at 4 °C. Sections were washed again with PBS, incubated with DAB Peroxidase (Vector Laboratories), and counterstained with hematoxylin. Slides were then mounted, and images were collected at 20X using an Olympus IX51 inverted microscope (Waltham, MA). Quantitative analysis was based on at least 110 images per group (*n* = 8 per group) using ImageJ software (NIH).

### Cultured primary islets

Primary mouse islets were isolated from male C57BL/6 mice (6–8 weeks old) using collagenase digestion as previously described [15], and human islets were provided by the NIDDK-funded Integrated Islet Distribution Program (IIDP) at City of Hope (NIH grant # 1UC4DK098085). Mouse and human islets, respectively, were separated into 30 mm dishes containing 50 islets per dish in DMEM (ThermoFisher Scientific) supplemented with 1% penicillin/streptomycin with 1% fatty acid free BSA (Sigma-Aldrich). Islets were cultured with high glucose (20 mM) or high glucose + palmitate (500 mM) in the presence or absence of CUR (1 nM) at 37 °C, 5% CO_2_ for 24 h.

### Quantitative real time-PCR

RNA was extracted using TRIzol (ThermoFisher Scientific) and a RNeasy Mini kit (Qiagen, Valencia, CA). Purified RNA (500 ng), an absorbance ratio of A260/A280 between 1.8 and 2.0, was converted into cDNA using qScript cDNA Supermix (Quanta Biosciences, Gaithersburg, MD) for mouse islets and SuperScript III First-Strand Synthesis System (ThermoFisher Scientific) for human islets. TXNIP gene expression was quantified using SYBR green (Quanta Biosciences) on an ABI Prism 7300 (Applied Biosystems, Foster City, CA) detection system and values were normalized to 18S. The mouse primers for TXNIP were forward: 5′-TCTTTTGAGGTCGTCTTCAACG-3′ and reverse: 5′-GCTTTGACTCGGTAACTTCACA-3’and the human primers were forward: 5′-ATATGGGTGTGTAGACTACTGGG-3′ and reverse: 5′-GACATCCACCAGATCCACTACT-3′ (Integrated DNA Technologies, Coralville, IA). Universal 18S primers were purchased from ThermoFisher Scientific.

### Statistical analysis

Body weight, food intake, water consumption, IPGTT, and ITT (%) were analyzed using two-way ANOVA repeated measure. Quantitative data are represented as the mean ± SEM. Quantification analysis for AUC, western blot band density and imaging pixels was conducted using one-way ANOVA followed by Tuckey’s multiple comparison after outlier test (α = 0.05). GraphPad Prism (Prism 7; GraphPad Inc.) was used to perform statistical analysis. *^, #^ *p* ≤ 0.05, **^, ##^ *p* ≤ 0.01, ***^, ###^ *p* ≤ 0.001, and **** *p* ≤ 0.0001 were considered statistically significant (* and ^#^ compared to control and HFS + CUR, respectively).

## Results

### Assessment of metabolic parameters of mice on diets

**Fig. 1:**
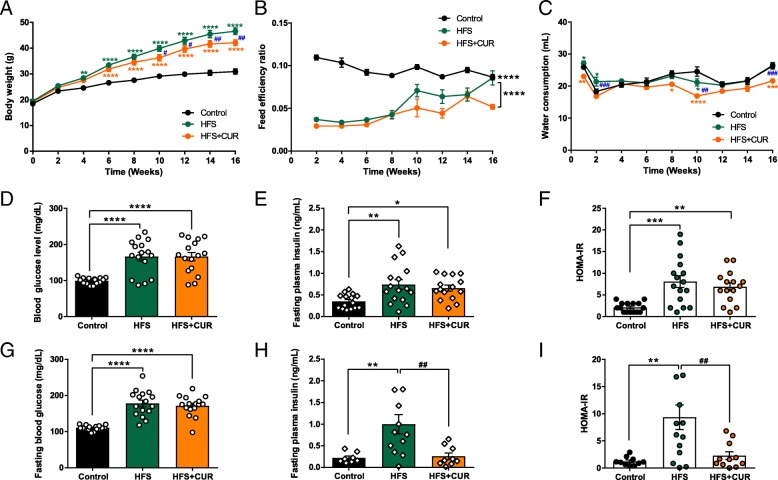
Curcumin (CUR) ameliorates obesity in high-fat/high-sugar (HFS) diet-induced obese mice. Control, HFS and HFS + CUR fed mice were measured biweekly for **a** Body weight (g), **b** accumulated food intake (g), and **c** water consumption (mL) (*n* = 16 per each group). Control, HFS, and HFS + CUR mice were fasted overnight, and blood glucose levels (mg/dL), plasma insulin levels (ng/mL) and HOMA-IR was calculated at 4 weeks (**d**-**f**) and at 16 weeks (**g**-**i**) of diet. (^*^p ≤ 0.05, ^**^p ≤ 0.01, ^***^p ≤ 0.001, ^****^p ≤ 0.0001 compared to Control; ^#^*p* ≤ 0.05, ^##^*p* ≤ 0.01, ^###^*p* ≤ 0.001 compared to HFS + CUR)

We found that C57BL/6 J mice (*N* = 16 per each group) administered a high fat/high sugar diet (HFS) for 16 weeks had a significant increase in body weight (46.5 ± 1.3 g) compared to those on a control diet (30.9 ± 1.0 g) (Fig. 1a). Surprisingly, mice administered a HFS diet containing 4 g/kg of curcumin (HFS + CUR) had significantly lower body weights (42.1 ± 1.1 g) compared to HFS diets alone from week 10 onwards. Both HFS and HFS + CUR fed mice had lower biweekly feed efficiency ratio compared to mice on a control diet, although there were no significant differences between each other (Fig. 1b). We found HFS + CUR fed mice overall tended to have less water consumption than the other groups (Fig. 1c).

After only 4 weeks on their respective diets, HFS and HFS + CUR fed mice had elevated fasting blood glucose (FBG; Fig. 1d) and serum fasting insulin (FI; Fig. 1e) levels compared to control diet mice (FBG: 166 ± 11.9, 166 ± 11.5, vs. 99 ± 2.2 mg/dL and FI: 0.74 ± 0.1, 0.66 ± 0.1, vs 0.35 ± 0.0 ng/mL). HOMA-IR, a method used to assess β-cell function and insulin resistance under fasting conditions, was also calculated and showed a significance between control and HFS supplemented groups (Fig. 1f). After 12 weeks on diets, we found FBG, FI, and HOMA-IR continued to rise in HFS mice, whereas HFS + CUR fed mice had lower levels by comparison (data not shown). At the end of the study, despite having similar FBG levels (178 ± 8.9 vs. 172 ± 7.0 mg/dL; Fig. 1g), HFS + CUR fed mice had lower FI levels (1.00 ± 0.2 vs. 0.26 ± 0.1 ng/ml; Fig. 1h) and HOMA-IR (9.3 ± 2.3 vs. 2.3 ± 0.7; Fig. 1i) compared to HFS mice. The lower values reflect increasing insulin sensitivity.

### CUR supplementation enhances insulin sensitivity and clearance in mice on HFS diet

**Fig. 2:**
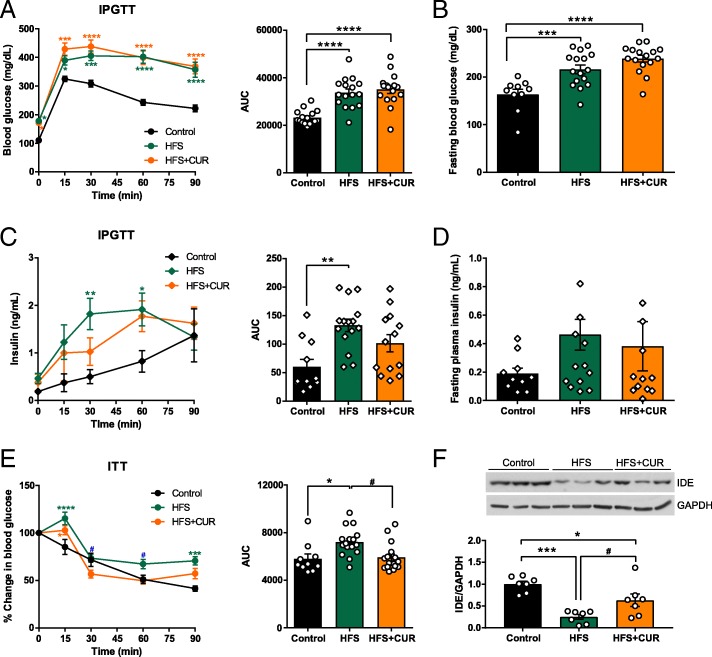
CUR treatment mitigates the development of insulin resistance in HFS diet fed mice. At week 16, Control, HFS, and HFS + CUR mice were fasted overnight and an intraperitoneal glucose tolerance test (IPGTT) was performed with1 g/kg of glucose. **a** Blood glucose levels (mg/dL) following IPGTT and the area under the curve (AUC) analysis. **b** Short-term (4 h) fasted blood glucose levels (mg/dL) at week 16. During IPGTT, blood samples were collected at each time point and **c** insulin levels (ng/mL) were measured and AUC calculated. **d** 4 h fasted insulin levels (ng/mL) at week 16. An insulin tolerance test (ITT) was performed 16 weeks after diet treatment (1 U/kg, i.p.). **e** The curve depicts the percentage values of initial blood glucose concentration and AUC during ITT. **f** IDE and GAPDH expression levels in liver tissue lysates on 16 weeks after diet treatment (*n* = 7) (^*^p ≤ 0.05, ^**^p ≤ 0.01, ^***^p ≤ 0.001, ^****^p ≤ 0.0001 compared to Control; ^#^*p* ≤ 0.05, ^##^*p* ≤ 0.01 compared to HFS + CUR)

HFS + CUR fed mice exhibited decreased FI levels with similar FBG levels as HFS mice, suggesting HFS + CUR fed mice were more insulin sensitive, so we performed an intraperitoneal glucose tolerance test (IPGTT). At week 16, HFS and HFS + CUR fed mice had significantly elevated blood glucose levels compared to control when blood glucose levels peaked at 30 min following an intraperitoneal injection of 1 g/kg glucose (406 ± 16.3, 438 ± 22.9 vs. 308 ± 11.7 mg/dL, Fig. 2a) under the prolonged (16 h) fasting condition. Moreover, HFS and HFS + CUR fed groups showed higher blood glucose levels than control group after short-term fasting (4 h) (Fig. 2b). When examining insulin secretion in response to glucose, HFS + CUR fed mice had similar insulin levels as HFS fed mice at baseline (0.38 ± 0.2 vs. 0.46 ± 0.1 ng/mL compared to control 0.19 ± 0.04 ng/mL), but significantly lower levels (1.0 ± 0.3 vs 1.8 ± 0.3 ng/mL compared to control 0.5 ± 0.2 ng/mL) 30 min after glucose administration (Fig. 2c). However, after 4 h fasting HFS fed mice had no significantly different insulin levels compared to control and HFS + CUR fed mice (Fig. 2d). In response to a bolus of insulin (1 U insulin/kg body weight), HFS + CUR fed mice were able to maintain their blood glucose levels in a similar manner to control mice, whereas HFS fed mice were significantly higher than others (Fig. 2e). Therefore, we sought to investigate how CUR administration leads to lower circulating insulin levels on high fat/high sugar diet challenge. We hypothesized HFS + CUR fed mice had increased insulin clearance compared to mice treated HFS alone. For this purpose, we analyzed the protein expression levels of hepatic insulin-degrading enzyme (IDE) and found that they were significantly reduced compared to control animals on HFS diets. Interestingly, HFS + CUR fed mice had partial restoration of IDE levels (Fig. 2f).

### CUR treatment alters liver morphology and function in hepatic insulin signaling pathway after administration of HFS diet

**Fig. 3:**
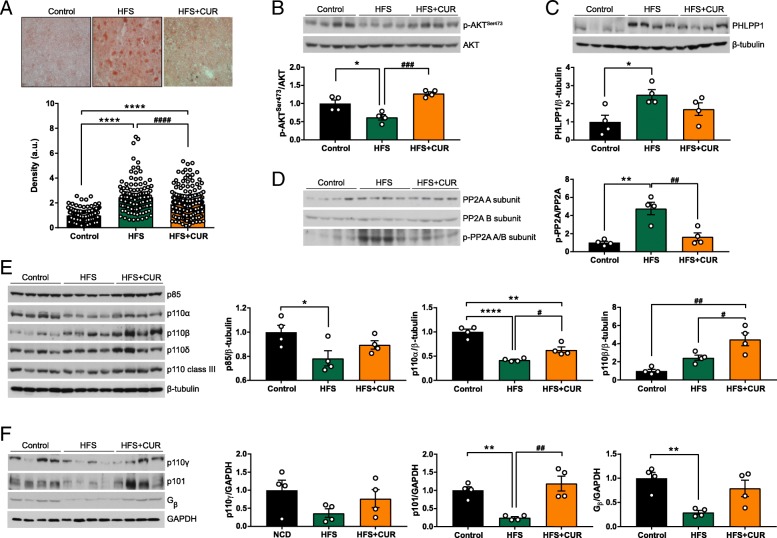
CUR administration reduces lipid accumulation and enhances downstream insulin signaling in the liver of HFS mice. Control, HFS and HFS + CUR fed mice were fasted overnight after 16 weeks of treatment. **a** Liver sections from Control, HFS and HFS + CUR fed mice were stained with Oil Red O and imaged at 20x objective. Quantitative analysis was based on at least 140 images/group (*n* = 8 mice per group). **b**-**f** Protein extracts from liver homogenates were immunoblotted and detected by autoradiographical signals. The bands were subjected to densitometric quantifications. **b** phospho-AKT^Ser473^ and total AKT analysis **c** PHLPP1 and β-tubulin **d** phosphor-PP2A A/B subunits and total PP2A A and B subunits **e** p85, p110α, β, or δ catalytic subunit, p110 class III and β-tubulin (*n* = 4 mice per group). **f** p110γ, p101, G_β_ and GAPDH (*n* = 4 mice per group). Integrated density was quantified using ImageJ software. Values were normalized to total AKT, PP2A A/B subunits, β-tubulin or GAPDH, respectively. (^*^p ≤ 0.05, ^**^p ≤ 0.01, ^****^p ≤ 0.0001 compared to Control; ^#^*p* ≤ 0.05, ^##^*p* ≤ 0.01, ^####^*p* ≤ 0.0001 compared to HFS + CUR)

Next, we examined the morphological and functional changes in the liver after 16 weeks administration of HFS diet. Liver sections of control, HFS, and HFS + CUR fed mice were stained with Oil Red O to analyze the relative amount of lipid accumulation. A 2.5-fold increase in lipid deposits in the liver of HFS fed mice compared to those given a control diet was observed. The density of staining for triglycerides and lipids was significantly reduced in the liver of HFS + CUR fed mice, despite being elevated compared to control, suggesting that CUR supplementation protects fatty liver in DIO mice (Fig. 3a).

Due to the fundamental role insulin plays in glucose and lipid metabolism in the liver, we investigated the expression of key nodes in the insulin signaling pathway. To do this, we first analyzed the phospho/total AKT in liver samples from control, HFS, and HFS + CUR fed mice. The values in HFS fed mice were significantly decreased compared to other groups. Surprisingly, the ratio of p-AKT/AKT in HFS + CUR fed mice is even higher than control group (Fig. 3b). Furthermore, to investigate whether CUR treatment regulates the specific phosphorylation of AKT, we detected pleckstrin homology domain (PH domain) and leucine rich repeat protein phosphatase 1 (PHLPP1) to dephosphorylate serine 473 site in AKT. Although HFS + CUR fed mice expressed slightly more PHLPP1 protein levels than control animals, HFS fed mice greatly upregulated PHLPP1, leading to less expression of p-AKT at Ser473 (Fig. 3c). In addition, another protein phosphatase to have serine/threonine phosphatase activity, protein phosphatase 2 (PP2A), was examined. HFS challenge activated phosphorylation of PP2A A/B subunit, however, CUR treatment ameliorated the expression levels of p-PP2A/B (Fig. 3d). We further sought to investigate whether CUR supplementation has modulatory effects on upstream regulators of AKT in the insulin signaling pathway. To prove this, we explored phosphoinositide 3-kinase (PI3K) composed of a regulatory and a catalytic subunit. We observed downregulation of PI3K class IA, a p85 regulatory subunit and p110α, β or δ catalytic subunit by HFS. On the other hand, CUR supplementation reinstated p110α and β protein levels in DIO mice (Fig. 3e). Moreover, HFS + CUR fed mice showed increased PI3K class IB, a p101 regulatory subunit and a p110γ catalytic subunit, activated by G protein-coupled receptors (GPCRs) via direct interaction with G beta-gamma complex (G_βγ_), and had 2.7-fold increase in G_β_ compared to mice treated HFS alone (Fig. 3f). Taken together, these results indicate that CUR treatment enhances insulin signaling via PI3K-AKT axis in the liver.

### CUR administration preserves normal islet integrity in mice on HFS diet

**Fig. 4:**
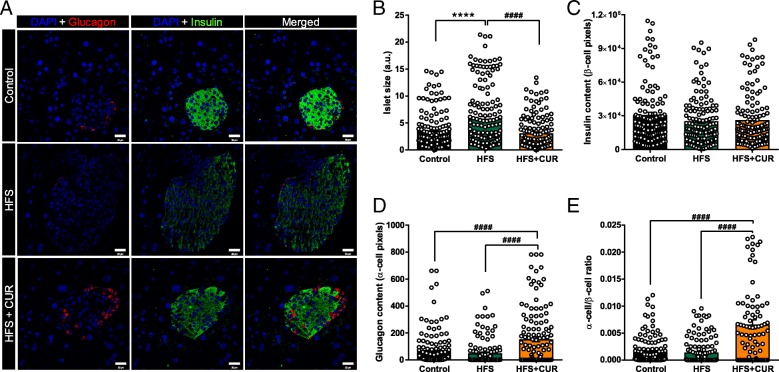
HFS + CUR fed mice maintain normal islet integrity. After 16 weeks of diet, **a** pancreas sections from mice on Control, HFS, or HFS + CUR diets were stained for insulin (green), glucagon (red), and DAPI (blue). Representative confocal images are shown using 40x oil objective. Pancreas^++^ software was used to quantitate **b** total islet size (scale bar, 20 μm), **c** insulin contents (β-cell pixels), **d** glucagon contents (α-cell pixels), and **e** ratio of α-cell/β-cell. Quantitative analysis was based on at least 150 images per group (*n* = 8 mice per group). (^****^p ≤ 0.0001 compared to Control; ^####^*p* ≤ 0.0001 compared to HFS + CUR)

Based on our glucometabolic data, CUR supplementation significantly enhanced insulin sensitivity. As a result, we examined the effects of CUR on the islets of Langerhans. We immunostained pancreata from control, HFS and HFS + CUR fed mice and measured islet size and relative α- and β-cell content after 16 weeks of diet (Fig. 4a). The islets from HFS fed mice (5.76 ± 0.44 a.u.) were markedly larger in size compared to islets of control diet fed mice (3.62 ± 0.31 a.u.). However, the administration of CUR to HFS fed mice preserved islet size (3.07 ± 0.25 a.u.) and resembled the islets of control diet mice (Fig. 4b), suggesting that CUR treatment may have enhanced insulin sensitivity by maintaining islet morphology. We also quantified intensity of labeled glucagon (α-cells) and insulin (β-cells) in the islets. Although HFS fed mice showed hyperplasia of β-cells, the insulin content in the islets was not different from other groups (Fig. 4c). Glucagon content was also increased in islets (Fig. 4d), and the overall ratio of α-cell/β-cell was significantly higher in HFS + CUR fed mice over control and HFS fed mice (Fig. 4e).

### CUR administration decreases TXNIP expression in islets of Langerhans during HFS conditions

**Fig. 5:**
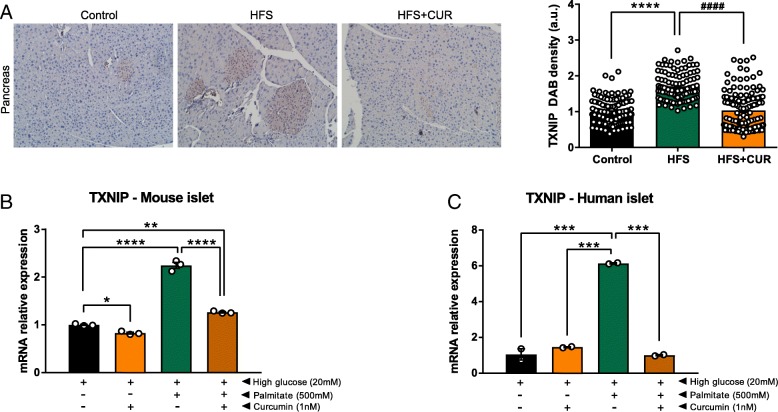
CUR administration decreases TXNIP expression. **a** At the end of the study, pancreas sections from Control, HFS, and HFS + CUR diet mice were DAB stained for TXNIP and quantified using ImageJ software. **b** and **c** Islets from C57Bl/6 J mice or human donors were cultured for 24 h under high glucose (20 mM) or high glucose plus palmitate (500 mM) conditions in the presence or absence of CUR (1 nM). TXNIP mRNA expression was determined by RT-PCR and normalized to 18S. (^**^p ≤ 0.01, ^***^p ≤ 0.001, ^****^p ≤ 0.0001 compared to each other)

One of the chief contributing factors to diet-induced obesity and the development of insulin resistance is the generation of oxidative stress [16]. Pancreatic islets lack the presence of antioxidant enzymes to counteract against stress. Therefore, we investigated the protective effects of dietary CUR on the protein TXNIP, which serves as a regulator of cellular metabolism and stress [17]. Although we found TXNIP to be ubiquitously expressed in pancreas tissue, its expression was greater in islets of Langerhans (Fig. 5a). Moreover, TXNIP density was 1.7-fold higher in islets of HFS fed mice, whereas HFS + CUR fed mice maintained a similar expression levels to control diet fed mice. Due to this significant change, we examined TXNIP mRNA expression in the isolated mouse and human islets cultured in high glucose and high glucose with palmitate conditions to mimic the HFS state (Fig. 5b and c). We found high glucose with palmitate conditions led to a significant increase in TXNIP expression, 2.2-fold in mouse and 6-fold in human, compared to high glucose alone. While CUR treatment did not appear to alter TXNIP expression under high glucose conditions, it dramatically inhibited TXNIP mRNA expression in high glucose with palmitate conditions, suggesting that CUR supplementation may exert its protective effects under “high fat” conditions.

## Discussion

In healthy individuals, β-cells are markedly plastic in their ability to regulate insulin levels on demand. To do this, they must maintain a continuous network of signaling with insulin-dependent tissues, such as liver, muscle, and adipose tissue [18]. In the current study, we examined the nutraceutical properties of dietary CUR in mice fed a continuous HFS diet. Dietary supplementation with CUR lowered body weight in mice when given a HFS diet, despite the fact that HFS + CUR fed mice had no difference in food intake compared to HFS fed mice. Fasting insulin levels are a primary indicator of insulin sensitivity. In the present study, we found that dietary CUR reduced the burden of diet-induced obesity in HFS mice by lowering circulating fasting insulin levels. Excess insulin during insulin-resistant states like T2DM deteriorates glucose and lipid metabolism, which lead to more fat accumulation via stimulation of lipogenesis [19].

The glucose levels in HFS and HFS + CUR mice were similar after long-term (16 h) fasting despite differences in fasting plasma insulin levels. This was most likely due to increased insulin resistance in HFS mice and decreased circulating insulin levels in HFS + CUR mice compared to HFS mice (Fig. 1g-i).

These metabolic parameters are systemically regulated by the networking of organs such as liver, skeletal muscle, and pancreas. There have been reports that rodents fed a high-fat diet for 12 months to induce obesity and insulin resistance displayed increased islet size and β-cell volume [20, 21]. Consequently, these increases were proposed to be a result of an increase in β-cell number as opposed to an actual change in β-cell size. Similarly, we found that islets from HFS fed mice were considerably larger in size compared to a control diet fed mice. Interestingly with CUR administration, islets maintained a normal size and phenotype and a significantly lower α-cell/β-cell ratio than other groups, indicating an ability to improve β-cell homeostasis.

Under normal physiological conditions, free fatty acids (FFA) are involved in gluconeogenesis and stored as triglycerides for long-term energy availability [22]. Increased circulating FFA levels, which can occur in obesity and T2DM, trigger the production of reactive oxygen species (ROS) [23], endoplasmic reticulum stress [24], and nuclear factor kappa B (NF-κB) transactivation [25]. Moreover, these factors act synergistically with glucose to induce glucolipotoxicity, which leads to deleterious effects on islet function and exacerbation of insulin resistance [26]. Based on previous studies, we know that therapeutics that aim to lower FFA levels can improve glucose uptake and insulin sensitivity [27]. In the current study, we found that CUR treatment was able to significantly regulate lipid accumulation in the liver compared to HFS diet alone.

Additionally, chronic exposure to elevated FFAs causes serine/threonine phosphorylation of insulin receptor substrate-(IRS)-1 and IRS-2, thus hindering the ability of these molecules to activate PI(3)K [28, 29]. Although in this study we cannot make a clear conclusion in terms of IR-IRS1-IRS2 axis in the insulin signaling pathway due to a variation of expression levels in mice within the same group (data not shown), we demonstrate CUR treatment restores PI3K levels, especially a regulatory p101 subunit and catalytic p110α and β subunits. Therefore, insulin signaling is more transduced by CUR supplementation, leading to positively regulated glucose-stimulated insulin secretion, insulin biosynthesis, and insulin clearance. Interestingly, here we demonstrate that reduced insulin degrading-enzyme (IDE) levels in HFS diet-induced obesity is a critical component in liver pathology. Our results show that CUR treatment reinstates IDE protein levels, and consequently improves insulin clearance.

Thioredoxin-interacting protein (TXNIP) is a key regulator and endogenous inhibitor of the thioredoxin system, which serves as a major scavenger for reactive oxygen species (ROS) [30]. More importantly, recent studies have implicated TXNIP as a potent contributor to the development of T2DM [31, 32]. For example, methylation of TXNIP was found to be inversely and intensely associated with HbA1c levels (≥7%) and explicitly corresponded to diabetic patients with poor control of glucose levels [31]. Other studies have determined that individuals who carry the T allele of the rs7211 polymorphism in the TXNIP gene present with higher plasma triglycerides levels, impaired glucose homeostasis, and increased risk of diabetes [33, 34]. Also, recent studies have shown that TXNIP induces oxidative stress and tissue damage through interaction with the NLRP3 inflammasome complex [35] as well as carbohydrate response element binding protein [36], which eventually lead to the deterioration and apoptosis of pancreatic β-cells [37]. In our study, dietary CUR administration prevented increased expression of TXNIP in pancreatic islets from HFS fed mice. Similarly, when primary mouse or human islets were cultured under HFS mimicking conditions, we found TXNIP mRNA expression increased 2-fold and 6-fold respectively, whereas CUR treatment prevented this occurrence. Although we did not address other oxidative stress markers except TXNIP with dietary CUR, this effect might be derived from decrease of oxidative stress by high fat diet mimicking condition [38]. Also, degradation of TXNIP upon energy stress by CUR treatment is dependent on AMP-dependent protein kinase (AMPK) pathway, leading to improved glucose homeostasis [39, 40]. Therefore, we further need to investigate the role of CUR in energy homeostasis-associated mechanisms to better understand how dietary CUR regulate circulating insulin levels.

## Conclusion

CUR supplementation in obese subjects may alleviate the burden imposed by HFS diets since dietary CUR enhances insulin clearance by restoring hepatic PI3K, AKT and IDE levels. Furthermore, CUR treatment maintains normal phenotype and function of islets of Langerhans, resulting from downregulation of TXNIP transcription levels. Dietary CUR may have the potential to serve as a novel therapeutic agent to address the underlying links of obesity and T2DM.

## Data Availability

Please contact author for data requests.

## Abbreviations

AUC: Area under the curve
CUR: Curcumin
DIO: Diet-induced obesity
FFA: Free fatty acids
GPCR: G protein-coupled receptor
G_βγ_: G beta-gamma complex
HFS: High fat high sugar
HOMA-IR: Homeostasis model for insulin resistance
IDE: Insulin-degrading enzyme
IPGTT: Intraperitoneal glucose tolerance test
IRS: Insulin receptor substrate
ITT: Insulin tolerance test
PH domain: Pleckstrin homology domain
PHLPP1: PH domain and leucine rich repeat protein phosphatase 1 (PHLPP1)
PI3K: Phosphoinositide 3-kinase
PP2A: Protein phosphatase 2
PVDF: Polyvinylidene fluoride
T2DM: Type 2 diabetes mellitus
TXNIP: Thioredoxin-interacting protein
